# Integrated Analysis of the LncRNA‐Mediated ceRNA Network Associated With Prognosis in Posttreatment Recurrent Nasopharyngeal Carcinoma

**DOI:** 10.1111/jcmm.70973

**Published:** 2025-12-07

**Authors:** Qihong Liu, Jingxiao Lu, Zhiqiang Wang, Qingming Li, Jianhui Wu, Kexing Lyu, Beiping Miao, Wenbin Lei, Guohui Nie, Xiaoqin Fan

**Affiliations:** ^1^ Department of Otolaryngology Head and Neck Surgery The Eighth Affiliated Hospital, Sun Yat‐sen University Shenzhen Guangdong P.R. China; ^2^ Shenzhen Key Laboratory of Nanozymes and Translational Cancer Research, Department of Otolaryngology, Shenzhen Institute of Translational Medicine The First Affiliated Hospital of Shenzhen University, Shenzhen Second People's Hospital Shenzhen Guangdong P.R. China; ^3^ The Bio‐Bank of Shenzhen Second People's Hospital The First Affiliated Hospital of Shenzhen University Shenzhen Guangdong P.R. China; ^4^ Department of Otolaryngology Zhongshan People's Hospital Zhongshan Guangdong P.R. China; ^5^ Department of Otorhinolaryngology Hospital, The First Affiliated Hospital Sun Yat‐sen University, National Key Department of Otorhinolaryngology of People's Republic of China Guangzhou Guangdong P.R. China

**Keywords:** ceRNA network, LncRNA sequencing, miRNA, nasopharyngeal cancer, relapse

## Abstract

The competing endogenous RNA hypothesis offers new insights into tumour progression, yet its role in posttreatment nasopharyngeal carcinoma relapse remains unclear. This study constructed ceRNA networks to identify molecular markers associated with NPC relapse. Three pairs of primary and relapse NPC tissue samples, along with their matched adjacent tissues, were collected for the RNA and miRNA sequencing, screened and identified relapse‐related specific differentially expressed genes. We identified relapse‐specific differentially expressed genes and functional analyses revealed enrichment in translation, biosynthesis, metabolism, TCA cycle, cell cycle, p53 signalling and immune pathways. Then these relapse‐associated differentially expressed mRNAs, lncRNAs, and miRNAs were utilised to construct regulatory networks, resulting in a ceRNA network comprising 813 mRNAs, 143 lncRNAs, and 24 miRNAs, along with a survival‐associated subnetwork of 23 mRNAs. Key mRNAs, such as UBC, PLA2R1, PTPRO, SMC5, PFN2, TIMM17B, NT5E and PCSK5, were validated via qPCR in NPC cell lines and tissues. To our knowledge, this is the first study to construct a comprehensive ceRNA network specifically for posttreatment recurrent NPC. These findings highlight the ceRNA network as a valuable framework for elucidating the mechanisms of NPC relapse and for identifying potential biomarkers for prognosis and therapeutic targets in recurrent nasopharyngeal carcinoma.

## 1 Introduction

Nasopharyngeal Carcinoma (NPC), a malignancy with high prevalence in southern regions of China and in Southeast Asia, is a distinct malignancy of the nasopharynx [1, 2]. Although the cure rate of NPC patients who are diagnosed as type I and II stage is more than 90% after radiotherapy or combined chemoradiotherapy, the significant rates of loco‐regional or distant relapse and metastasis of type III and IV stage, and approximately 30% of NPC patients suffer relapse and metastasis after comprehensive therapy [3]. Due to the insufficient comprehension of the molecular interactions and the complex relapse process, the clinical treatment and management of the relapse post‐treatment NPC is still facing largely challenges [4]. Thus, there remains an urgent need for finding the potential molecular interaction biomarkers and developing novel targets for predicting the post‐treatment relapse process.

In the competing endogenous RNA (ceRNA) theory, various researches integrate the transcriptome to construct large‐scale ceRNA networks to predict the progression of disease. A comprehensive post‐transcriptional regulatory network highlights the role of long non‐coding RNAs (lncRNAs), a type of non‐protein‐coding RNA with transcripts exceeding 200 nucleotides, in regulating gene expression at both transcriptional and post‐transcriptional levels [5]. Over the last decade, increasing studies have found that the lncRNAs have contributed to the epigenetic modifications, translational regulation, post‐translational modifications and protein/mRNA stability influenced through interactions with DNA, RNA and protein [6]. More and more functional studies indicated that lncRNAs are closely linked to subcellular localisation and the transcriptional loci of the genes they regulate. Based on the previous studies, lncRNAs participated in the onset and progression of multiple cancers, including Breast Cancer [7], Lung Cancer [8], Hepatocellular Carcinoma [9], and Nasopharyngeal Carcinoma [10]. As such, these networks offer a framework for understanding intricate gene interactions and identifying potential biomarkers to enhance the clinical diagnosis and management of NPC.

Many lncRNAs were dysregulated and played important roles in the onset, progression, relapse and metastasis of NPC. A number of studies have shown that lncRNAs could act as either oncogenes or tumour suppressor genes by regulating mRNAs through various mechanisms [11]. For example, the lncRNAs played the role of oncogene, such as PVT1 [12], RP11‐624L4.1 [13], AFAP1‐AS1 [14], DANCR [15] and ANCR [16]. Whereas other lncRNAs produce an effect of tumour suppressor, such as ZNF667‐AS1 [17], MEG3 [18] and LET [19]. Although some studies have investigated the roles and mechanisms of lncRNAs in NPC, the majority primarily focus on their onset and progression [20, 21, 22, 23, 24, 25]. So, the role and mechanism of biological roles driven by dysregulation of lncRNAs in relapse‐posttreatment of NPC remain in‐depth scarce.

The cellular functions of lncRNAs were indicated as important regulators in gene expression networks especially by binding to other non‐coding RNA or super enhancer [26]. In NPC, lncRNA‐FAM225A modulated integrin beta3 (ITGB3) expression to promote NPC cell proliferation and invasion by targeting miR‐590‐3p and miR‐1275 mediated by the activation of the FAK/PI3K/Akt signalling pathway [27]. LncRNA SUCLG2‐AS1 was found to be located in the super enhancer region of SOX2, and regulates the expression of SOX2 via long‐range chromatin loop formation, which regulates the metastasis and radiosensitivity of NPC [28]. Thus, previous research has shown that lncRNAs are key regulators of NPC progression through their influence on mRNAs, and their relative role in NPC requires further investigation. Although the ceRNA hypothesis has been extensively explored in primary nasopharyngeal carcinoma [29, 30, 31, 32, 33, 34, 35], leading to the identification of several oncogenic or tumour‐suppressive lncRNA‐miRNA‐mRNA axes, these studies have predominantly focused on tumorigenesis and initial progression. While emerging studies have begun to characterise the molecular landscape of recurrent NPC using approaches such as single‐cell sequencing and transcriptomic analyses [36, 37, 38, 39, 40, 41, 42, 43, 44, 45, 46, 47], the comprehensive architecture and specific role of ceRNA networks in post‐treatment recurrence remain completely uncharted territory. This critical knowledge gap significantly hinders our understanding of the post‐transcriptional regulatory drivers of relapse and the development of RNA‐based targeted strategies to prevent or treat it. To address this limitation, our study employs an integrated multi‐omics approach to specifically construct and characterise the first recurrence‐specific ceRNA network in NPC.

More recently, the whole transcriptome sequencing of various cancers has fundamentally performed our understanding of the underlying genomic panorama and molecular basis of cancers, and more recently has provided approaches to characterise and monitor cancers in the clinic, guiding and treatment. Herein, we compared the significantly differentially expressed mRNAs, lncRNAs and miRNAs between the relapse posttreatment and primary NPC tissues through the whole transcriptome sequencing. This approach uniquely positions our study to address the critical gap in knowledge regarding posttreatment recurrence, as existing ceRNA networks in NPC have been largely derived from primary, untreated tumours. In addition, Gene Ontology (GO) and Kyoto Encyclopedia of Genes and Genomes (KEGG) analysis, and the target miRNAs were predicted and crossed to obtain their target mRNAs. Then lncRNA‐miRNA‐mRNA regulator networks were established using the significantly differentially expressed RNAs. The primary objective was to construct a relapse‐specific ceRNA landscape, which remains entirely unexplored compared to the well‐investigated networks in primary NPC. Finally, the survival‐related mRNAs in this ceRNA network were further validated as a potential biomarker by using the NPC cells and NPC tissues in vitro experiments.

## 2 Methods

### 2.1 Clincial Specimens

Primary NPC tissues and paired adjacent samples from three patients were collected from the Department of Head Neck Surgery, Otolaryngology, Shenzhen Second People's Hospital during 2022 (Table 1) [48]. Additionally, three pairs of relapse‐posttreatment samples from patients including NPC and adjacent tumour‐free tissues were collected at the Department of Otolaryngology, The Eighth Affiliated Hospital of Sun Yat‐sen University, Shenzhen Futian District People's Hospital between 2022 and 2023 (Table 1). To minimise technical variability and potential batch effects, all freshly collected tissue specimens were immediately snap‐frozen and stored using standardised protocols until RNA extraction. Crucially, RNA extraction, library preparation, and sequencing for all samples were performed simultaneously in the same batch under identical conditions. According to the sample criteria, the adjacent tumour‐free tissues were collected about 5 mm distant from the tumorous tissues. The diagnoses of relapse posttreatment tissues and the nearby adjacent tissues were independently verified by histopathology. All patients provided written informed consent, and the study was ethically approved by the committee of the Eighth Affiliated Hospital of Sun Yat‐sen University (No. 2022‐029‐01). The samples were surgically resected and immediately preserved in RNA‐later.

**TABLE 1:** Patient clinical information.

Patient ID	Gender	Age	Race	Province	Prior treatment	Collection date	Primary diagnosis	Diagnosis is primary disease
Primary NPC 1	Male	46	East Asian	Guangdong	No	2022‐04‐02	Nasopharynx carcinoma	True
Primary NPC 2	Female	30	East Asian	Guangdong	No	2022‐05‐21	Nasopharynx carcinoma	True
Primary NPC 3	Male	38	East Asian	Guangdong	No	2022‐07‐01	Nasopharynx carcinoma	True
Relapse NPC 1	Female	43	East Asian	Guangdong	Yes	2022‐03‐20	Nasopharynx carcinoma	False
Relapse NPC 2	Male	58	East asian	Guangdong	Yes	2022‐08‐10	Nasopharynx carcinoma	False
Relapse NPC 3	Male	55	East asian	Guangdong	Yes	2023‐01‐06	Nasopharynx carcinoma	False

### 2.2 Histopathological Analysis

Haematoxylin and eosin (H&E) staining was used to assess the tumour area. The specimens were initially deparaffinised with Histo‐Clear II reagent (National Diagnostics, USA) then rehydrated through a graded alcohol series. The sections were performed following the standard protocol of H&E staining.

### 2.3 RNA Sequencing

A total of 2 mg fresh tissues were obtained for each sample. Total RNA was extracted from the tissues using TRIzol (Invitrogen, USA) according to the manufacturer's instructions. The quantity and purity of each sample were analysed by using Nano Drop (ABI, USA) with RNA integrity number (RIN) > 7. The high quantity RNA was used to construct libraries for RNA‐sequencing. Library construction and sequencing were performed and finally sequenced using Illumina nova 6000 platforms of Novogenes (Beijing, China).

### 2.4 Small RNA Sequencing

Total RNA was extracted using TRIzol reagent. miRNA sequencing libraries were constructed with the NEBNext Multiplex Small RNA Library Prep Kit. Sequencing was performed on an Illumina NovaSeq 6000 platform with 50 bp single‐end reads.

### 2.5 Differential Expression Analysis of mRNAs and lncRNAs

The raw fastqs were evaluated using fastp (v0.20.1) and fastqc (v0.11.9) tools for quality assessment, and adapter sequences were removed using the trim‐galore tool (v 0.4.4_dev). The clean data were then mapped to the hg38 genome using hisat2 software (v2.2.1), and transcript expression quantification was performed using the stringtie software (v2.1.4) to obtain gene FPKM expression data. Additionally, we used featureCounts (v2.0.1) to quantify gene counts from bam files to get the raw count Matrix for genes. To assess the relationships between samples, PCA analysis was performed on the FPKM expression files to check the reliability of the sample data and the clustering of different groups. In order to evaluate the relationship between the samples, PCA analysis were performed on the FPKM expression Matrix to check the reliability of the sample data and the clustering of different groups. Differential expression analysis of the mRNAs and lncRNAs in primary and relapse specimens between the NPC group and the adjacent group were used by DESeq2 package (v1.38.3) in R 4.2.1 (http://bioconductor.org/packages/DESeq2/) [49]. The significant screening criteria of |log2FC| ≥ 1 and *p*‐value ≤ 0.01 were accepted for differentially expressed mRNAs and differentially expressed long noncoding RNAs. We visualised the differentially expressed mRNAs and lncRNAs using the heatmap and volcano plot by pheatmap and ggplot2 packages, respectively.

### 2.6 Differential Expression Analysis of miRNAs

For primary NPC data, Human miRNA Array‐based datasets GSE32960 and GSE36682 were downloaded from the GEO database, which contained 18,6 controls and 312,62 primary NPC tissues, respectively. We used limma tool to analyse the differential expression miRNAs in the normalised expression profiles of miRNA. The dataset GSE118720 was based on the Illumina HiSeq platform and included 4 controls and 7 primary NPC tissues. We used the DESeq2 tool for differential analysis of the miRNA count matrix. For recurrent NPC data, we performed quality control on the sequencing data of 3 pairs of recurrent NPC samples collected by our group use fastp tools (v0.20.1). Then bowtie software (v1.3.0) was used to filter out rRNA, tRNA and other sequences, and miRNA clean data were retained. After all of this, known miRNAs were quantified by mirdeep2 software (v0.1.2), and the miRNA count expression matrix was obtained, the differential miRNAs was analysed by DESeq2 software. For significantly analysis in GEO database, *p*‐value < 0.05 and |Fold Change| > 1.5 were used to identify significantly differential expressed miRNAs. In the data of relapse NPC, we used *p*‐value < 0.05 and |Fold Change| > 2 as significant screening criteria. In addition, for miRNA functional analysis, we utilised miTarbase, TarBase and ENCORI databases to predict target genes, and further used the obtained target genes for miRNA functional enrichment analysis and correlation network construction.

### 2.7 Enrichment Analysis of Differentially Expressed mRNAs and lncRNAs

Differentially expressed mRNAs and lncRNAs were then utilised to explore and enrich biological features, such as biological processes, cellular components, molecular functions, and associated key pathways. Moreover, Gene Ontology (GO) and Kyoto Encyclopedia of Genes and Genomes (KEGG) enrichment analysis were used to identify the significant pathways by GOseq and Kobas3 software. *p*‐value < 0.05 was considered statistically significant. For the functional analysis of lncRNAs, we used cis‐regulatory methods to obtain target genes and thus predict their functions. To explore the activity status of different samples on different pathways, we scored the pathway activity of all pathways in the “hallmark” genesets collection using the GSVA package. In order to explore the pathway activity of each sample, we used the GSVA package (v1.46.0) to calculate the activity score for all pathways in the hallmark gene sets from the MSigDB database. Moreover, the Wilcox test was used to examine the statistically significant differences in pathway activity among groups.

### 2.8 Protein–Protein Interaction Network and Module Construction

The interaction network of key genes, including both direct and indirect protein interactions and their functional correlations, was constructed using the NetworkAnalyst online tool and STRING database. Molecular interactions and PPI networks facilitate the exploration of the molecular functions underlying relapse posttreatment in NPC. Therefore, the differentially expressed mRNAs were used to construct the PPI network. Then the Mcode package in Cytosape software (v3.9.1) was used to extract the key hub nodes from the PPI networks.

### 2.9 Survival Analysis

The dataset of GSE102349 which is based on Illumina HiSeq was downloaded from the Gene Expression Omnibus (GEO) database (https://www.ncbi.nlm.nih.gov/geo/). This dataset consisted of genome‐wide expression data, including 113 NPC tissues, of which only 88 samples had available prognostic information. The survival curves of all genes were then analysed using the Kaplan–Meier method with the survival package, and the results were tested using the log‐rank method.

### 2.10 Construction of lncRNA‐Mediated ceRNA Regulate Networks

We obtained differential lncRNA‐associated target genes using the cis‐regulate method, and additionally obtained differential miRNA‐associated target genes using the miRNA target gene database including miRTarBase and TarBase, prioritising experimentally validated interactions. Then, based on the competitive regulatory mechanism of ceRNAs and integrating lncRNA‐mRNA, mRNA‐miRNA and lncRNA‐miRNA data, we successfully constructed the differential lncRNA‐mediated ceRNA regulatory network and visualised the ceRNA network using Cytoscape software.

### 2.11 The Key RNAs Validation by Q‐PCR

Base on the bioinformatics analysis of DEGs in the relapse NPC group, we validated the expression level of 8 differentially expressed mRNAs, which were selected from the 23 survival‐associated hub genes based on a multi‐tiered criterion (including |log2FC| ≥ 1, *p*‐value ≤ 0.05, prognostic value ≤ 0.05, basal FPKM expression level ≥ 1, and the top network topology), in vitro NPC cell models and NPC tissues by Q‐PCR. Total RNA was extracted from different cell lines with TRIzol Reagent (Invitrogen, USA) according to the manufacturer's instructions. The extracted total 2 μg RNA of every cell line sample was reverse transcribed with PrimeScript RT Reagent Kit (Takara, Japan) and amplified by using SYBR Green Realtime PCR Master Mix Kit (Takara, Japan). The relative expressional levels of differentially expressed mRNAs were evaluated and normalised to GAPDH by using the 2^−ΔΔCT^. Sequences of all primers are provided in Table 2.

**TABLE 2:** The primers of the Q‐PCR.

Number	Name	Species	Primers (forward and reverse primer)
1	GAPDH	Human	F: 5′GGAGCGAGATCCCTCCAAAAT‐3′
			R: 5′GGCTGTTGTCATACTTCTCATGG‐3′
2	NT5E	Human	F: 5′CCAGTACCAGGGCACTATCTG‐3′
			R: 5′TGGCTCGATCAGTCCTTCCA‐3′
3	PLA2R1	Human	F: 5′GCATACAATCAAAGGGAACACCC‐3′
			R: 5′TCGTGGCACACCACAGTAAG‐3′
4	SMC5	Human	F: 5′TCATGGGACGAGCAGATAAGG‐3′
			R: 5′TCACGGGTGATTACAAGATTTCC‐3′
5	UBC	Human	F: 5′CTGGAAGATGGTCGTACCCTG‐3′
			R: 5′GGTCTTGCCAGTGAGTGTCT‐3′
6	TIMM17B	Human	F: 5′ATGGAGGAGTACGCTCGGG‐3′
			R: 5′CCGATGACACCCATAGTGAAGG‐3′
7	PTPRO	Human	F: 5′ATGACTTCAGCCGTGTGAGAT‐3′
			R: 5′ GGGTGGCAATATACTCCTGGG‐3′
8	PCSK5	Human	F: 5′GAGGGACCCACAGTTTCATTTC‐3′
			R: 5′TGGGCACGACTGAAGTCATAA‐3′
9	PFN2	Human	F: 5′ATGATTGTAGGAAAAGACCGGGA‐3′
			F: 5′GCAGTCACCATCGACGTATAGAC‐3′

### 2.12 Statistical Analysis

All experiments were performed in triplicate, and the data are presented as the mean ± standard deviation (SD). Statistical analysis was carried out using Student's *t*‐test in GraphPad Prism 7 software with significance levels defined as *p < 0.05 and **p < 0.01, respectively. The pheatmap, and ggplot2 packages were used for visualisation of results.

## 3 Result

### 3.1 The Histological Alteration of ICH in Relapse of NPC

**FIGURE 1:**
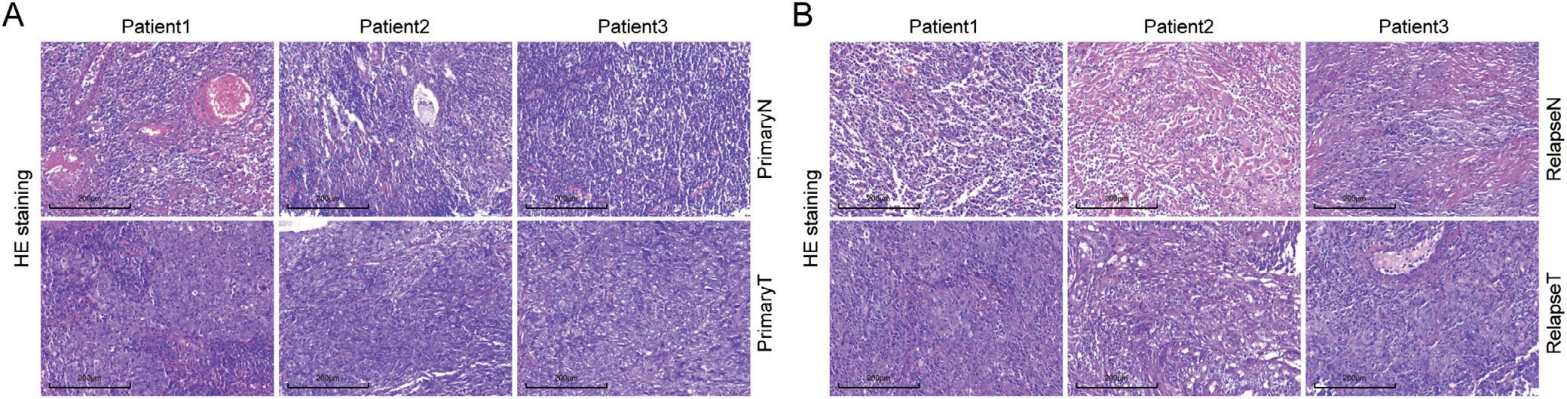
HE staining image of NPC tissues. (A) Representative HE staining image of primary NPC and NT tissue. (B) Representative HE staining image of relapse‐posttreatment of NPC and NT. NPC, relapse nasopharyngeal carcinoma tissues; NT, adjacent non‐tumorous tissues. Scar bar: 250 μm.

Post‐treatment relapse in NPC patients presents a substantial challenge for clinical therapies. To identify candidate key molecular biomarkers for post‐treatment relapse, we conducted whole‐transcriptome sequencing analysis. The study population comprised 3 NPC patients with primary tumours and 3 NPC patients with relapsed tumours. The detailed clinical histological alteration information of the three pairs of primary NPCs and adjacent tissues was displayed in Figure 1A. Furthermore, the detailed clinical histological changes of relapsed NPCs and adjacent tissues were shown in Figure 1B.

### 3.2 DE mRNAs Identification in the Primary and Relapse NPCs

**FIGURE 2:**
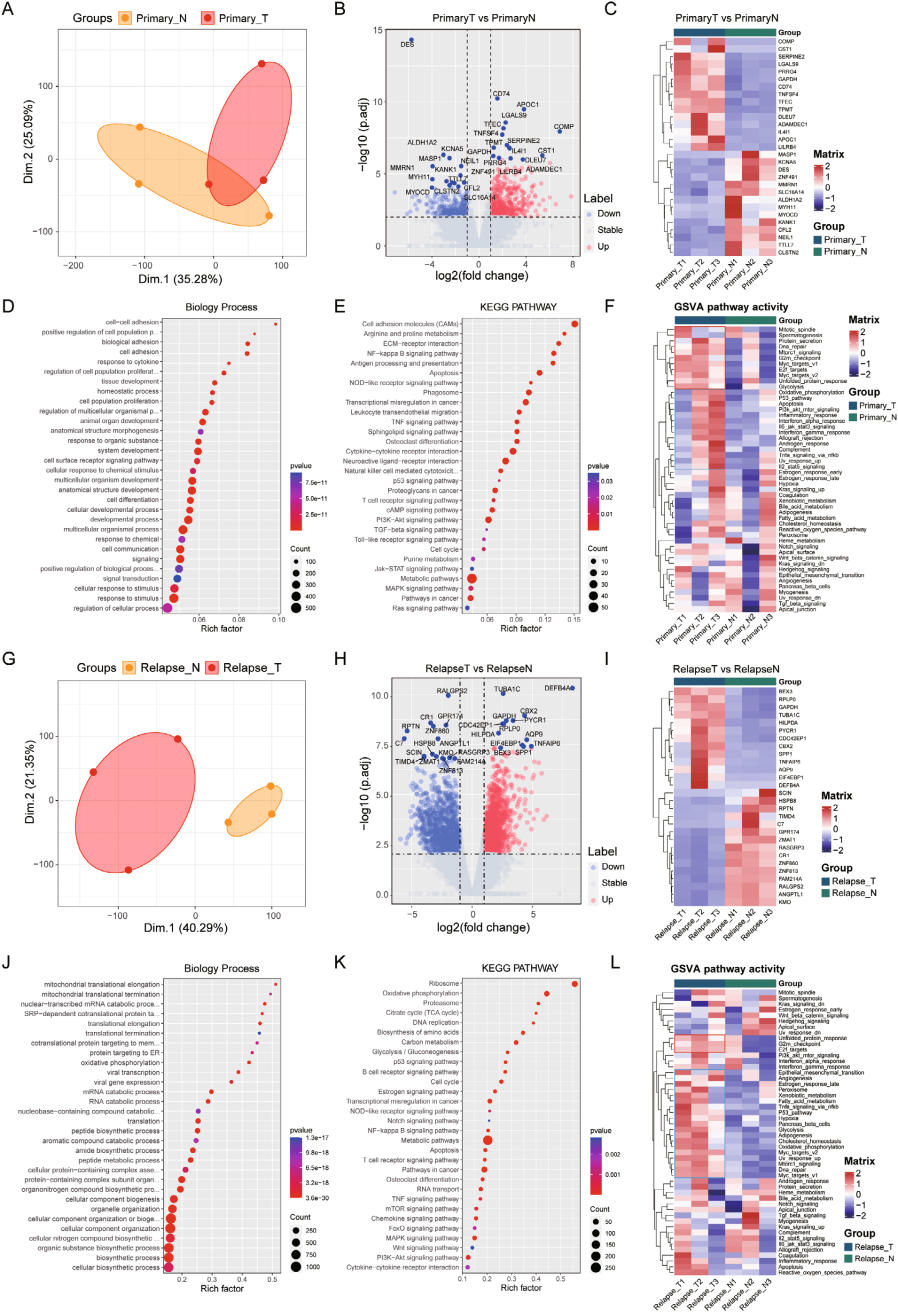
Differentially expressed mRNAs analysis of the NPC primary and relapse posttreatment groups. (A) The PCA map of the NPC primary group; (B) Volcano map of differentially expressed mRNAs in the primary group; (C) Heatmap of top 15 up/down‐regulated differentially expressed mRNAs in the primary group; (D) Bubble graph of differentially expressed mRNAs enriched by GO analysis in the primary group; (E) Bubble graph of differentially expressed mRNAs enriched by KEGG function analysis in the primary group; (F) Heatmap of GSVA enrichment using hallmark genesets in the primary group; (G) The PCA map of the NPC relapse group; (H) Volcano map of differentially expressed mRNAs in the relapse group; (I) Heatmap of top 15 up/down‐regulated differentially expressed mRNAs in the relapse group; (J) Bubble graph of differentially expressed mRNAs enriched by GO analysis in the relapse group; (K) Bubble graph of differentially expressed mRNAs enriched by KEGG function analysis in the relapse group; (L) Heatmap of GSVA enrichment using hallmark genesets in the relapse group.

To investigate the pathogenesis of nasopharyngeal carcinoma progression and identify the key genes that play a role in progression in both primary and recurrent NPC, we conducted RNA‐Seq analysis on the tissues of both primary and recurrent NPC and their adjacent normal tissues. First, we performed PCA analysis on the primary NPC tissues and their matched adjacent samples (Figure 2A), which showed that primary nasopharyngeal cancer tissues and their paired adjacent samples had their respective expression profiles. Then, DESeq2 was used to analyse the differential expression of primary NPC samples. A total of 765 differentially expressed mRNAs were identified in primary NPCs, with 478 genes up‐regulated and 287 genes down‐regulated (Figure 2B, Table S1), the top 15 most significantly changed gene expressions are shown as heatmaps (Figure 2C), where we can see that the COMP, CST1, PRRG4, GAPDH, CD74, TNFSF4, TFEC, TPMT, DLEU7, ADAMDEC1, IL4L1, APOC1, LIRB4 genes are significantly up‐regulated in the primary NPCs, while the MASP1, KCNA5, DES, ZNF491, MMRN1, SLC16A14, ALDH1A2, MYH11, MYOCD, KANK1, CFL2, NEIL1, TTL17 CLSTN2 genes are significantly down‐regulated. In addition, we performed functional enrichment analysis of 765 differential genes, which showed that the biological processes such as cell adhesion‐related pathways and development‐related pathways were significantly enriched (Figure 2D), and KEGG signalling pathways such as “Cell adhesion molecules”, “ECM‐receptor interaction”, “NF‐kappa B signalling pathway”, “Apoptosis”, “NOD‐like receptor signalling pathway” and “Osteoclast differentiation” were also significantly enriched (Figure 2E, Table S2). Furthermore, we analysed the pathway activity of primary NPCs by GSVA method, and we found that compare with adjacent samples, the proliferation‐related and immune‐related signalling pathways were significantly activated in primary NPC tissues (Figure 2F), which indicated that the primary NPC tissues had significant microenvironmental alterations. Similarly, PCA analysis was also performed on recurrent NPC tissues, and it was found that there were significant differences between recurrent NPC tissues and adjacent samples (Figure 2G). DESeq2 differential analysis on relapsed NPC showed that a total of 2707 differential mRNAs were identified, among which 1566 differential genes were up‐regulated and 1141 were down‐regulated in relapsed NPC tissues (Figure 2H, Table S3), the top 15 significantly differential genes expression were shown in the heatmap (Figure 2I). As a result, we can see that BEX3, RPLP0, GAPDH, TUBA1C, HILPDA, PYCR1, CDC42EP1, CBX2, SPP1, TNFAIP6, AQP9, EIF4EBP1, DEFB4A gene is significantly up‐regulated in recurrent NPC tissue, while SCIN, HSPB8, RPTN, TIMD4, C7, GPR174, ZMAT1, RASGRP3, CR1, ZNF860, ZNF813, FAM214A, RALGPS2, ANGPTL1, KMO gene is significantly down‐regulated. Function enrichment analysis showed that the biological process such as mitochondrial‐related pathway and biosynthetic‐related pathways was significantly enriched (Figure 2J), and “Oxidative phosphorylation”, “DNA replication”, “p53 signalling pathway”, “B cell receptor signalling pathway” and “Cell cycle” pathway was significantly enriched in KEGG pathways (Figure 2K, Table S4). GSVA analysis showed that proliferation‐related, DNA damage‐related, and metabolic‐related signalling pathways were significantly activated in recurrent NPC tissues (Figure 2L). This finding indicates substantial alterations in the microenvironment of recurrent NPC tissues compared to adjacent normal tissues and suggests that the progression mechanisms of recurrent NPC differ from those of primary NPC.

### 3.3 Identification of the Significant Differentially Expressed mRNAs in Relapse Posttreatment NPC

**FIGURE 3:**
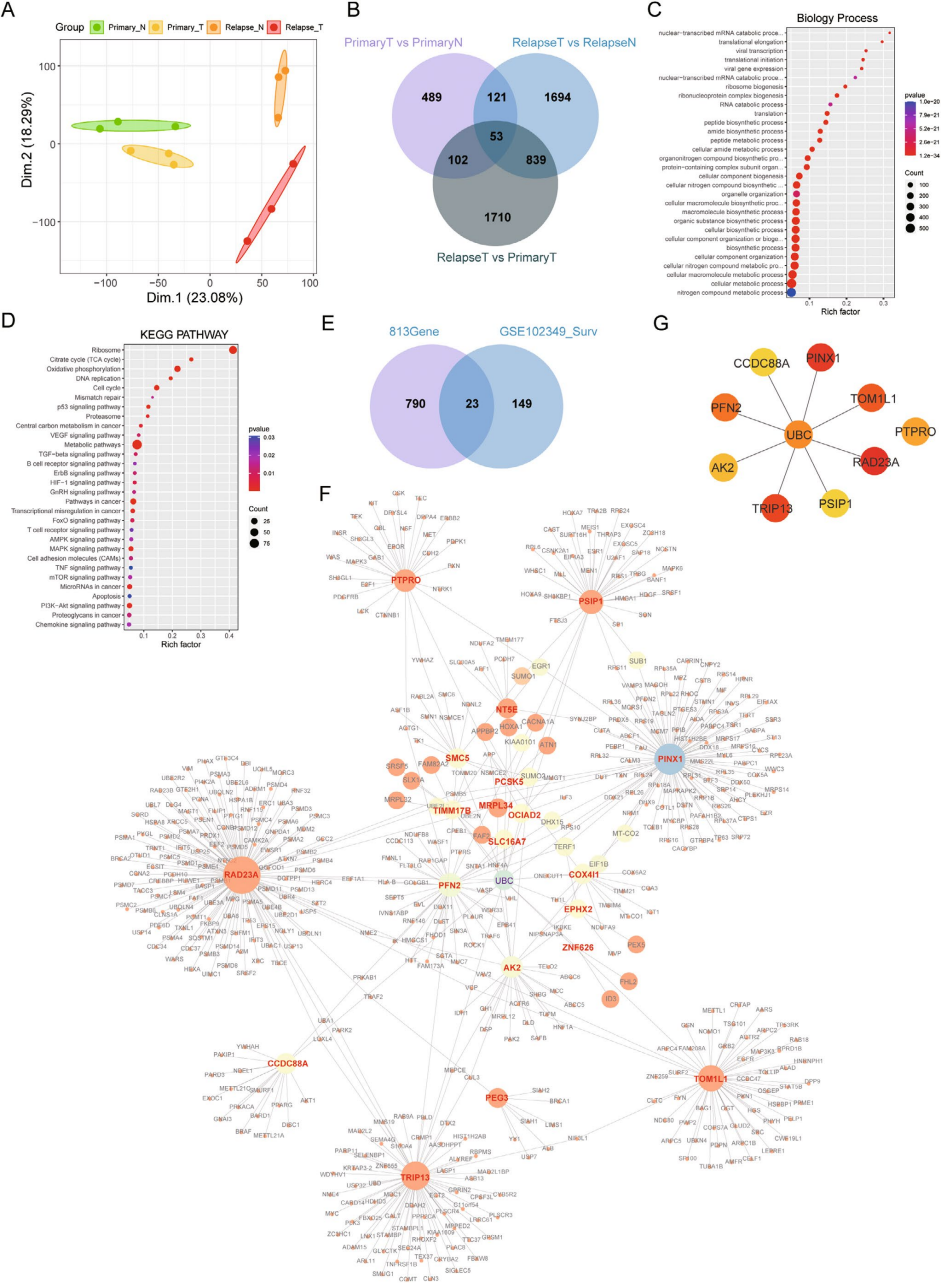
Differentially expressed mRNAs analysis of the relapse posttreatment NPC. (A) The PCA map of mRNA in the NPC primary and relapse groups; (B) Venn diagram shows that the significant differentially expressed mRNAs of the relapse in NPC; (C) Bubble graph of the relapse‐specific 839 differentially expressed mRNAs enriched by GO analysis in the NPC relapse; (D) Bubble graph of the relapse‐specific 839 differentially expressed mRNAs enriched by KEGG analysis in the NPC relapse; (E) Venn diagram of the significant differentially expressed mRNAs of the survival prognosis in the relapse in NPC and the GSE102349; (F) Protein–protein interaction (PPI) and TF analysis with 23 hub genes; (G) Protein–protein interaction analysis with UBC associated with the 10 core genes.

In order to explore the specific differentially expressed mRNAs during nasopharyngeal recurrence, we performed PCA analysis on primary and recurrent samples, and significant differences were observed between the samples of different groups, as shown by the results (Figure 3A). In order to obtain the key mRNAs specific to relapse, we first performed a differential analysis between the recurrent NPC samples and the primary NPC samples. The results showed that, compared with the primary NPC samples, there were a total of 2704 differentially expressed mRNAs, among which 1312 genes were up‐regulated, 1392 genes were down‐regulated (Figure S1A, Table S5), and the expression of the top 15 genes such as KRT4, EIF3CL, FBL, SLC34A2, MUC16, CEACAM5 SFRP4, COMP, FOS, GAS1, TFEC, TMED7 with the most significant changes were as shown in the heatmap (Figure S1B). Biological Process enrichment results showed that “biological synthesis”, “metabolism process” and translational‐related processes were significantly enriched (Figure S1C), and KEGG analysis showed that signalling pathways such as “p53 signalling pathway”, “Cell cycle”, “Carbon metabolism”, “TGF‐beta signalling pathway”, “Osteoclast differentiation” and “PI3K‐Akt signalling pathway” were significantly enriched (Figure S1D, Table S6). Then, we screened the results of the 3 differential analyses using a Venn diagram, which showed that there were 53 nasopharyngeal cancer differential genes in the 3 groups, and we guessed that these 53 genes significantly promoted the progression process of nasopharyngeal cancer. Meanwhile, we found a total of 839 key genes specific for NPC relapse, so we guessed that these 839 genes significantly contributed to the relapse process of nasopharyngeal cancer (Figure 3B, Table S7). In order to understand the key factors of NPC relapse, we conducted a functional enrichment analysis on these 839 mRNAs, and the results showed that biological processes such as translational‐related processes, biosynthetic‐related processes and metabolic‐related processes were significantly enriched (Figure 3C), and KEGG pathways such as “TCA cycle”, “Oxidative phosphorylation”, “DNA replication”, “Mismatch repair” and “Proteasome” were also significantly enriched (Figure 3D, Table S8). In order to distinguish the up‐ and down‐regulated regulatory features, we screened the up‐ and down‐regulated differential genes in each group separately, in which in the up‐regulated screen we obtained 578 molecules that are key to the up‐regulation of NPC relapse (Figure S1E), and the KEGG enrichment analysis results showed that the “Ribosome”, “Metabolic pathways”, “Oxidative phosphorylation”, “Spliceosome” and “Cell cycle” were significantly enriched (Figure S1F). In the down‐regulated screen we obtained 235 molecules that are key to the down‐regulation of NPC relapse (Figure S1G), and the enrichment analysis results showed that “Choline metabolism in cancer”, “B cell receptor signalling pathway”, “VEGF signalling pathway”, “Osteoclast differentiation” and “Cell adhesion molecules” were significantly enriched (Figure S1H). In addition, in order to screen the key mRNAs significantly associated with nasopharyngeal cancer recurrence from 839 genes, we performed survival analysis using the GSE102349 dataset. A total of 23 genes demonstrated a significant association with overall survival (Figure 3E). Kaplan–Meier survival analysis validated the prognostic utility of these genes (Figure S1I,J). Furthermore, we constructed protein interaction networks and transcription factor regulatory networks for these 23 key genes, and analysed the key sub‐network molecules using the MCODE tool. The analysis results showed that there were complex regulatory relationships among these relapse‐related genes (Figure 3F). Among them, the UBC gene, identified as a key regulatory molecule with significant prognostic, exhibits close regulatory relationships with multiple partners including RAD23A, PSIP1, TRIP13, AK2, PFN2, CCDC88A, PINX1, TOM1L1, and PTPRO (Figures 3G and S1J), suggesting it may play a crucial role within the network. Nevertheless, despite being strongly associated with NPC relapse, the functional mechanisms of UBC require further in‐depth investigation.

### 3.4 Differentially Expressed lncRNAs Identification in the NPC Primary and Relapse Groups

**FIGURE 4:**
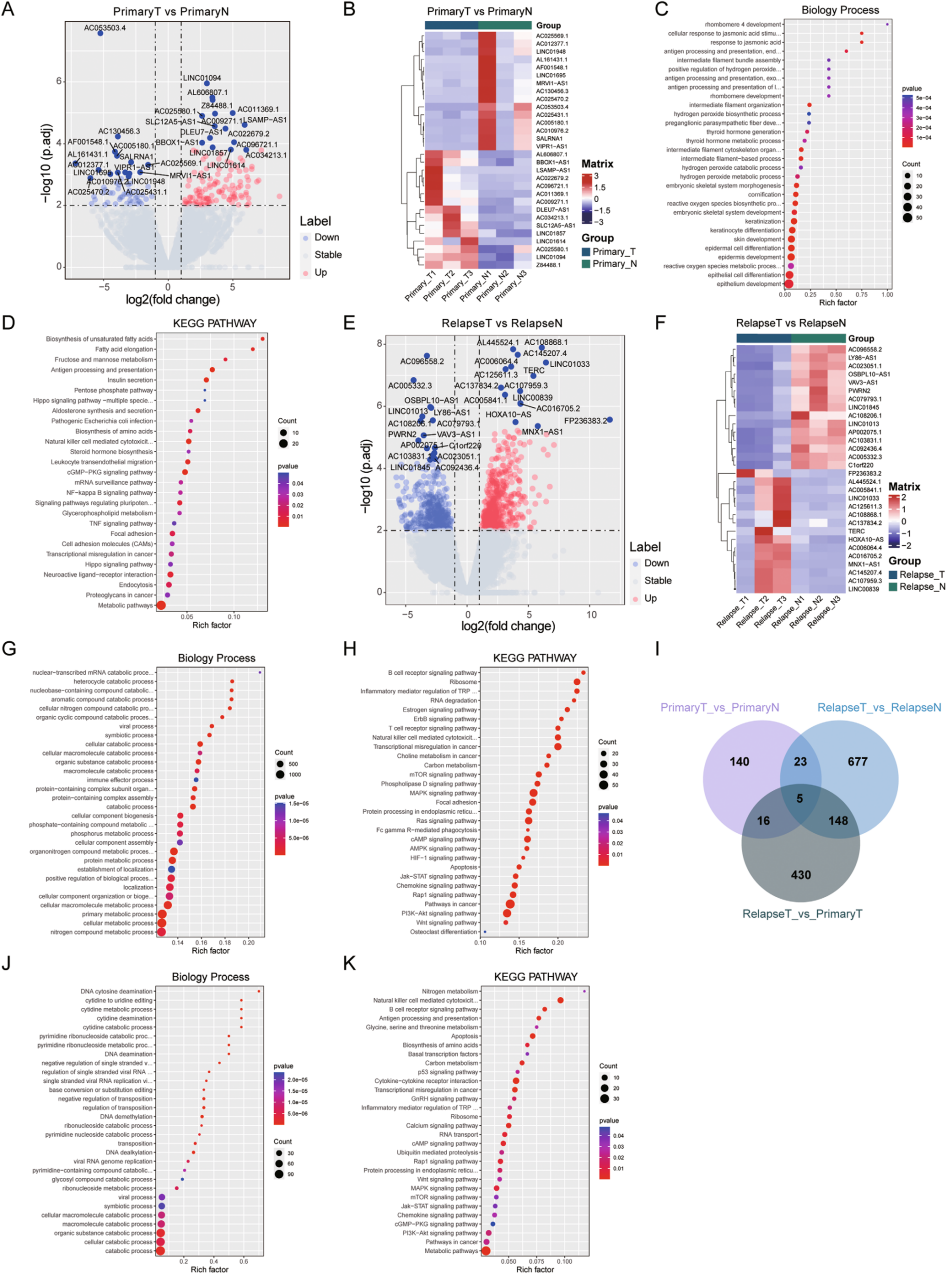
Differentially expressed lncRNAs analysis of the relapse posttreatment in NPC. (A) Volcano map of differentially expressed lncRNAs in the primary NPC group; (B) Heatmap of top 15 up/down‐regulated differentially expressed lncRNAs in the primary NPC group; (C) Bubble graph of differentially expressed lncRNAs enriched by GO analysis in the primary NPC group; (D) Bubble graph of differentially expressed lncRNAs enriched by KEGG function analysis in the primary NPC group; (E) Volcano map of differentially expressed lncRNAs in the relapse NPC group; (F) Heatmap of top 15 up/down‐regulated differentially expressed lncRNAs in the relapse NPC group; (G) Bubble graph of differentially expressed lncRNAs enriched by GO analysis in the relapse NPC group; (H) Bubble graph of differentially expressed lncRNAs enriched by KEGG function analysis in the relapse NPC group; (I) Venn diagram of the significant differentially expressed lncRNAs of the relapse in NPC; (J) Bubble graph of the relapse‐specific significant differentially expressed lncRNAs enriched by GO analysis; (K) Bubble graph of the relapse‐specific significant differentially expressed lncRNAs enriched by GO analysis.

To explore the regulatory role of lncRNA in the relapse of NPC, and screen out the key regulatory molecules related to relapse, we analysed the lncRNA data of primary and recurrent NPC samples. First, differential analysis of primary NPC samples showed that among the 8534 lncRNAs, 184 lncRNAs exhibited significant differential expression, including 129 up‐regulated and 55 down‐regulated (Figure 4A, Table S9). The expression of the top 15 genes such as AL606807, AC022679, DLEU7‐AS1, SLC12A5‐AS1, LINC01857, Z84488 and AC025569, LINC01948, LINC01695, AC005180, ASLRNA1, VIPR1‐AS1 with the most significant changes was shown as a heat map (Figure 4B). Functional enrichment analysis demonstrated that biological process was mainly enriched in development‐related and epithelium‐related processes (Figure 4C), while the KEGG pathway was mainly enriched in “Biosynthesis of unsaturated fatty acids”, “Fatty acid elongation”, “Natural killer cell mediated cytotoxicity”, “Steroid hormone biosynthesis” and “Endocytosis” (Figure 4D, Table S10). Subsequently, the differential analysis of relapse NPC samples showed that among 8930 lncRNAs, 853 lncRNAs showed significant differential expression, including 494 up‐regulated and 359 down‐regulated (Figure 4E, Table S11). Similarly, the expression of the top 15 genes such as AL445524, LINC01033, AC108868, TERC, HOXA10‐AS, MNX1‐AS1 and LY86‐AS1, PWRN2, LINC01845, LINC01013, AC005332, C1orf220 with the most significant changes was displayed in the heatmap (Figure 4F). Functional enrichment analysis showed that biological process was mainly enriched in processes such as catabolic‐related processes and metabolic‐related processes (Figure 4G), while KEGG pathway was mainly enriched in “B cell receptor signalling pathway”, “Ribosome”, “T cell receptor signalling pathway”, “Transcriptional mis‐regulation in cancer” and “Osteoclast differentiation” signalling pathways (Figure 4H, Table S12). In addition, to explore the differences between recurrent NPC and primary NPC, we performed DEseq2 difference analysis on these two groups of samples, and found that 599 lncRNAs were significantly different, of which 250 were up‐regulated and 349 were down‐regulated (Figure S2A, Table S13), the top 15 genes such as AC122108, GPRC5D‐AS1, LINC02081, RMRP, AC011294, FAM3D‐AS1 and LINC00472, AC124016, LINC01291, AC007336, DPYD‐AS2, CLMAT3 with the most significant changes in expression are shown in the heatmap (Figure S2B). Functional enrichment analysis showed that biological process was mainly enriched in processes such as glucuronidation‐related processes, transposition‐related processes and development‐related processes (Figure S2C), while the KEGG pathway was similarly enriched in “Ascorbate and aldarate metabolism”, “B cell receptor signalling pathway”, “Fatty acid degradation”, “Transcriptional mis‐regulation in cancer” and “Pathways in cancer” signalling pathways (Figure S2D, Table S14). In order to identify key lncRNAs in NPC relapse, we conducted Venn diagram screening on the genelist results of the above three groups, and found that there were 5 common lncRNA genes (AL022724, AC006064, TERC, AC025569, AC130456) differential expression in the three groups, which may have a pivotal role in NPC progression. Meanwhile, there were 148 lncRNAs specific to nasopharyngeal cancer relapse, suggesting that these lncRNA genes may be key to NPC recurrence (Figure 4I, Table S15). Functional enrichment analysis of these 148 relapse‐specific lncRNAs showed that the biological process was mainly enriched in processes such as transposition‐related and catabolic‐related processes (Figure 4J), while KEGG pathway was mainly enriched in pathways such as “Nitrogen metabolism”, “Apoptosis”, “Biosynthesis of amino acids”, “p53 signalling pathway” and “Cytokine‐cytokine receptor interaction” (Figure 4K, Table S16). In addition, we identified 114 genes with up‐regulated expression and 29 genes with down‐regulated expression among 148 lncRNAs (Figure S2E,F).

### 3.5 Identification of the Significant Differentially Expressed miRNAs for NPC Relapse

**FIGURE 5:**
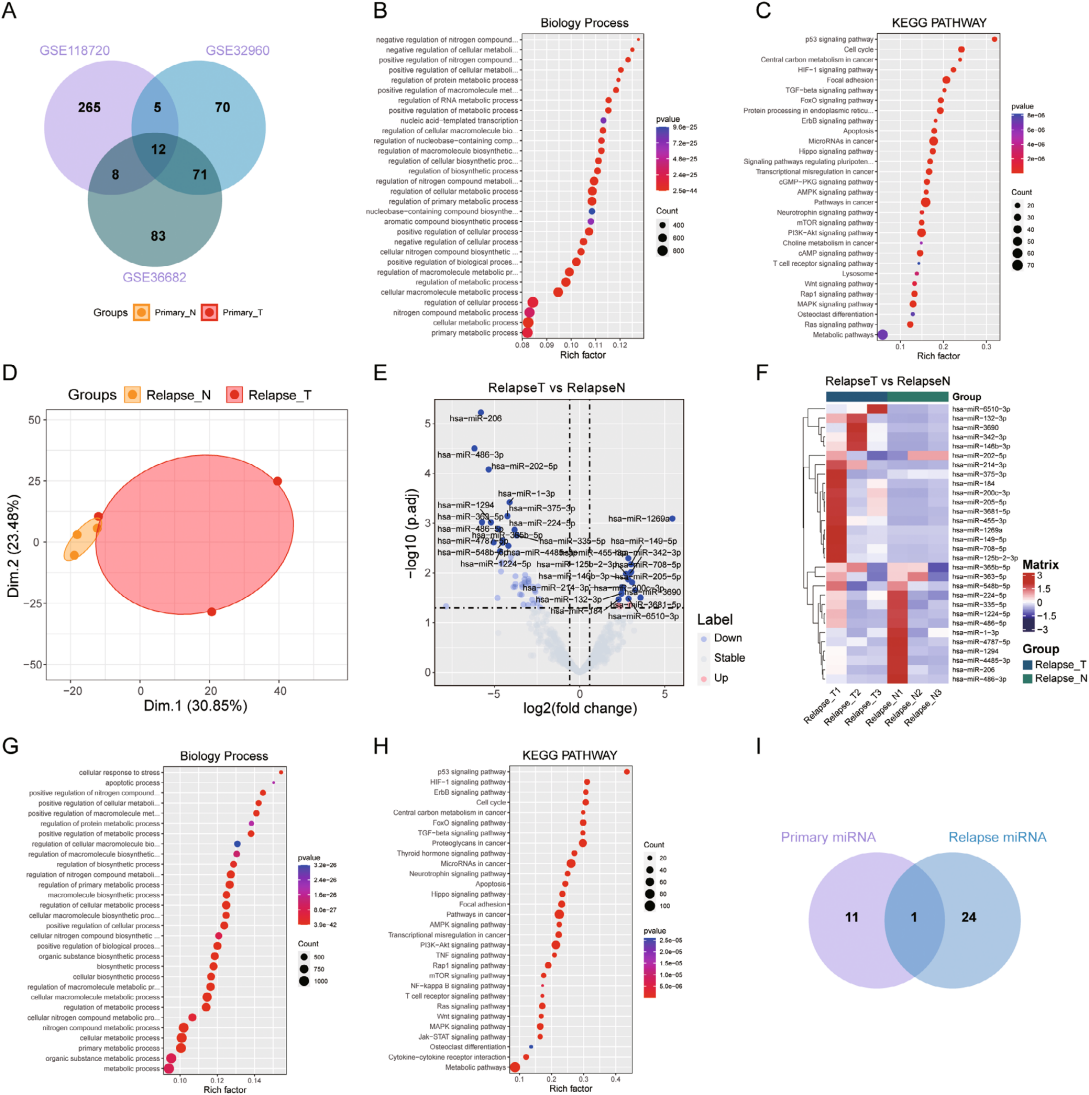
Differentially expressed miRNAs analysis of the relapse in NPC. (A) Venn diagram of 12 differentially expressed miRNAs in the three NPC primary tissues public datasets; (B) Bubble graph of the 12 differentially expressed miRNAs enriched by GO analysis; (C) Bubble graph of 12 differentially expressed miRNAs enriched by KEGG function analysis; (D) The PCA map of miRNAs in the NPC relapse group; (E) Volcano map of differentially expressed miRNAs in the relapse group; (F) Heatmap of the top 15 up/down‐regulated differentially expressed miRNAs in the relapse group; (G) Bubble graph of the target genes of differentially expressed miRNAs enriched by GO analysis in the relapse group; (H) Bubble graph of the target genes of differentially expressed miRNAs enriched by KEGG function analysis in the relapse group; (I) Venn diagram of the primary top 12 differentially expressed miRNAs and relapse miRNAs.

To explore the regulatory role of miRNAs in NPC recurrence and to identify key miRNA regulatory molecules, we performed a systematic analysis of NPC‐related miRNA data. First, for the miRNA analysis of primary NPC, we downloaded three datasets, GSE118720, GSE32960 and GSE36682, which included NPC primary tissues and paired adjacent tissues from publicly available datasets, and performed differential expression analysis respectively. The results showed that 291, 161, and 182 differential miRNAs were detected, among which 155, 75, and 94 miRNAs were up‐regulated, and 135, 86, and 88 miRNAs were down‐regulated (Figure 5A, Tables S17–S19). Further screening by Venn diagrams revealed 12 common miRNAs from these three datasets, which may play a significant regulatory role in the progression of NPC. In order to further understand the functions of these 12 miRNAs, we predicted their target genes using miTarBase and TarBase databases, and performed functional enrichment analysis of target genes. The results showed that the biological processes were mainly enriched in processes such as regulation‐related processes and compound‐related processes (Figure 5B), while KEGG pathway was significantly enriched in “p53 signalling pathway”, “Cell cycle”, “HIF‐1 signalling pathway”, “Focal adhesion” and “Osteoclast differentiation” (Figure 5C, Table S20). These findings align with the results of the previous mRNA functional enrichment analysis, indicating that these miRNAs significantly influence the progression of nasopharyngeal cancer by regulating the expression of related mRNAs. For the analysis of recurrent nasopharyngeal cancer samples, we first performed miRNA quantification and PCA analysis on the sequencing data, which showed that the recurrent tumour samples exhibited large heterogeneity, which might be related to the individual differences of patients (Figure 5D). Subsequently, we performed DESeq2 difference analysis between recurrent NPC tissues and paired adjacent tissues, identifying 25 differential miRNAs, of which 4 were up‐regulated and 21 were down‐regulated (Figure 5E, Table S21). The expression of the top 15 miRNAs such as hsa‐miR6510‐3p, hsa‐miR‐132‐3p, hsa‐miR‐3690 and hsa‐miR‐206, has‐miR‐1294, hsa‐miR‐1‐3p with significant differences was shown in the heatmap (Figure 5F). Functional enrichment analysis showed that the biological processes of these 25 miRNAs were mainly enriched in apoptotic process and metabolic‐related processes and biosynthetic‐related processes (Figure 5G), while the KEGG pathway was significantly enriched in “p53 signalling pathway”, “HIF‐1 signalling pathway”, “ErbB signalling pathway”, “Cell cycle” and “Osteoclast differentiation” (Figure 5H, Table S22). To further screen out the key miRNA for NPC relapse, we filtered primary miRNAs using a recurrent miRNA gene list and found that the hsa‐miR‐342‐3p gene was present in both primary and recurrent samples, suggesting that hsa‐miR‐342‐3p was significantly contributes to the progression of nasopharyngeal cancer. In addition, the remaining 24 miRNAs were specific to NPC relapse, indicating that these 24 miRNAs be crucial in the progression of NPC recurrence (Figure 5I, Table S23).

### 3.6 lncRNA‐miRNA‐mRNA (ceRNA) Regulated Networks Analysis and qPCR Verification

**FIGURE 6:**
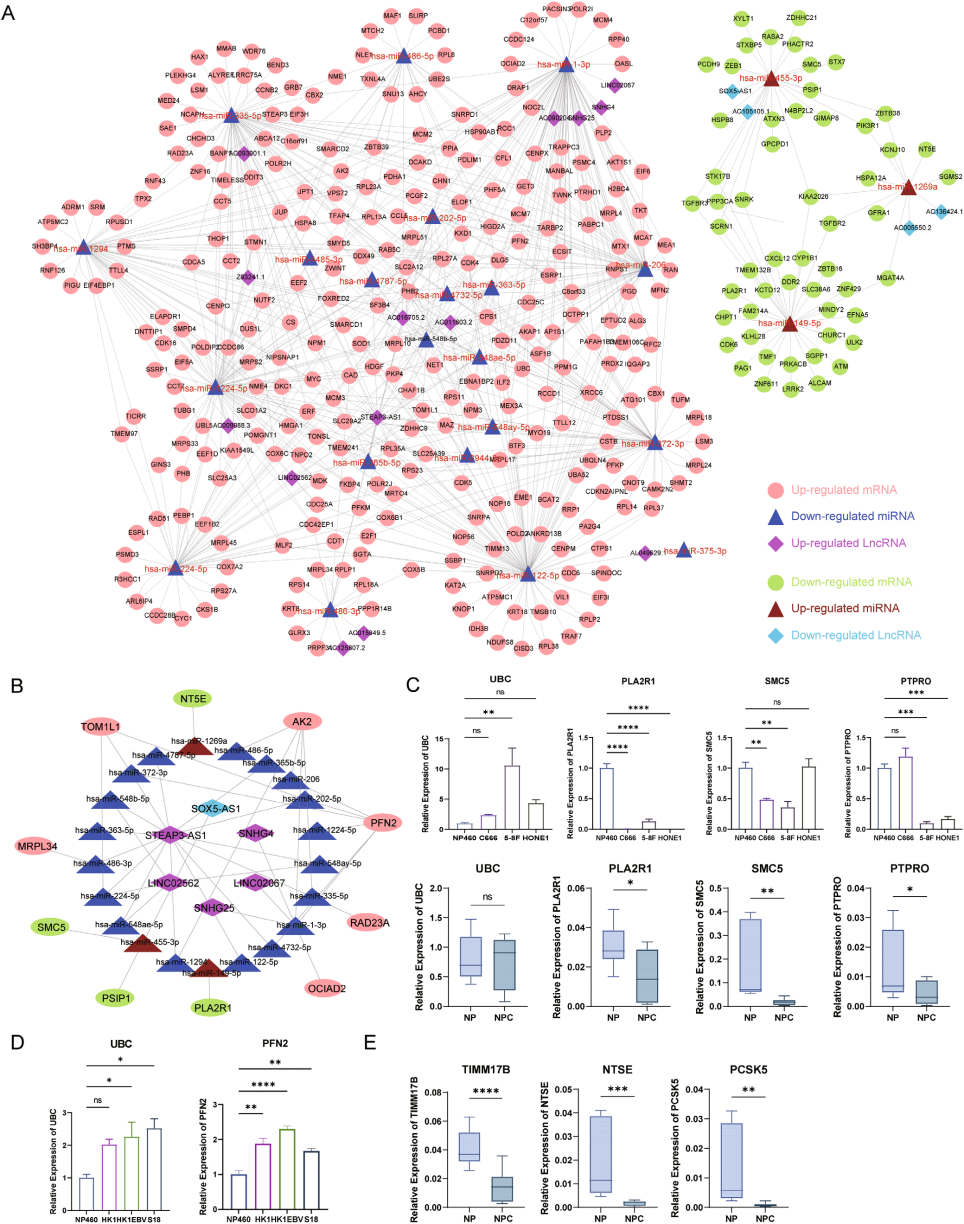
Construction and validation of co‐expressional networks. (A) CeRNA network constructed using significant differentially expressed lncRNAs, miRNAs and mRNAs; (B) CeRNA network constructed by 23 survival‐related mRNAs; (C) QPCR validation of 4 mRNAs in NPC cell lines and paired NPC tissues; (D) QPCR validation of the UBC and PFN2 genes in other NPC cell lines; (E) QPCR validation of 3 mRNAs in paired NPC tissues; *p < 0.05, **p < 0.01, ***p < 0.001, ****p < 0.0001.

In order to further understand the regulatory relationship of competitive endogenous RNA (ceRNA) in the process of nasopharyngeal cancer recurrence and explore its potential molecular mechanism, we constructed a ceRNA network based on the specific genes related to nasopharyngeal cancer relapse and their competitive regulatory relationship. Finally, a total of 813 mRNAs, 143 lncRNAs and 24 miRNAs constituted a complex ceRNA regulatory network, which consisted of 387 nodes and 739 edges (Figure 6A, Table S24). These RNA molecules interact through competitive regulatory relationships and constitute an important regulatory framework for NPC relapse. To further screen key ceRNA regulatory molecules with survival significance, we constructed a survival‐specific ceRNA subnetwork based on 23 significant mRNAs. The results show that the network contains 70 nodes and 169 edges, and these survival‐associated ceRNAs may regulate the recurrence process of NPC through this competitive approach (Figure 6B, Table S24). These ceRNAs may exert an important influence on NPC recurrence.

To verify the reliability of our data, we selected 4 of the most significantly differentially expressed mRNAs from 23 mRNAs based on their significant fold change and prognostic value, then performed qPCR validation in NPC cell lines (C666, 5‐8F, HONE1) and primary NPC patient tissues. The results showed that compared with the normal group, genes such as PLA2R1, SMC5, and PTPRO were significantly differentially expressed (Figure 6C), consistent with the RNA sequencing data. However, the UBC expression did not differ significantly in clinical samples. To further corroborate these findings, we validated the expression of UBC and PFN2 in three additional NPC cell lines (HK1, HK1‐EBV, S18). Both genes showed significant upregulation in these models and were also consistent with the RNA sequencing result (Figure 6D). Furthermore, we additionally selected 3 significantly differentially expressed mRNAs for validation in primary NPC patient tissues. Among these, 3 genes including TIMM17B, NTSE and PCSK5 were significantly down‐regulated in tumour tissues compared to normal controls, which is consistent with prior sequencing results (Figure 6E). All these results suggest that the ceRNA network for nasopharyngeal cancer recurrence and the regulatory molecules of this network are vital in disease progression, and also highlight the importance of further validation experiments to clarify its consistency and reliability across different models.

## 4 Discussion

With the development of precise therapies for NPC, many new technologies and markers of epigenetics especially lncRNAs [50] and circRNAs [32], which could function as regulators of host gene expression in the pathological process of NPC, are good choices in targeted therapy. Therefore, the endogenous RNAs (ceRNAs), especially the significant expression of lncRNA‐miRNA‐mRNA networks underlying the predisposition of relapse posttreatment of NPC urgently need further study. The transcriptome sequencing (RNA‐seq) is a powerful and well‐established approach for analysing the full transcriptome [51]. In our earlier work, we examined the interplay between mRNA and circRNA in primary NPC by integrating RNA sequencing with bioinformatics analyses [48]. To address the clinically significant challenge of posttreatment recurrence in NPC, we employed whole‐transcriptome sequencing to identify significantly dysregulated mRNAs and lncRNAs in recurrent NPC tissues. To our knowledge, this is the first study to construct a comprehensive ceRNA network specifically for posttreatment recurrent NPC. Our integrated analysis not only reveals key regulatory RNAs but also highlights the involvement of immune and metabolic pathways in NPC recurrence. By constructing a lncRNA–miRNA–mRNA regulatory network and identifying hub genes, this work provides novel molecular insights and potential therapeutic avenues for precise treatment of NPC relapse.

The co‐expression‐regulated ceRNA network hypothesis suggests that lncRNAs, mRNAs, and other RNA types can regulate and influence tumour recurrence and metastasis [52]. The co‐expressional ceRNA regulatory networks could better explain the interaction among different types of RNAs at the genetic and epigenetic level. Earlier research has explored the involvement of ceRNAs in tumour recurrence and metastasis in various types of cancer [29, 31, 33, 34, 35, 41]. The lncRNA HOXA‐AS2 acts as a ceRNA for miR‐184 to regulate the expression of COL6A2 to involve the recurrence and metastasis of glioma through constructing the ceRNA regulator networks of lncRNAs, miRNAs, and mRNAs by comprehensive transcriptomic analysis of multiple datasets from CGGA and TCGA datasets [53]. In Head and Neck Squamous Cell Carcinoma (HNSCC), 79 lncRNAs86 86 miRNAs, and 324 mRNAs were identified by integrated bioinformatics analysis based on the TCGA datasets [23]. Despite exploring the lncRNA‐miRNA‐mRNA regulatory networks in various cancers, these studies have not comprehensively examined the co‐expressional ceRNA networks in relapsed NPC. In this study, we systematically analysed transcriptome sequencing data to identify relapse‐related differentially expressed mRNAs and lncRNAs, and then built the ceRNA regulatory network for relapse in NPC. In our study, we found there were 813 differentially expressed mRNAs, and 23 key mRNAs with survival prognosis by using the GSE102349 dataset, which had the prognostic information to analyse in the relapse NPC group respectively. Meanwhile, 143 relapse‐associated differentially expressed lncRNAs were also identified. Three primary NPC miRNA array datasets (GSE118720, GSE32960 and GSE36682) were used to screen the candidate target miRNAs and 24miRNAs were found. We selected the differentially expressed mRNAs, lncRNAs and candidate miRNAs to build the regulator networks for the relapse of NPC.

To further validate the prognostic relevance of the identified survival‐associated genes, we selected 8 significantly differentially expressed mRNAs from 23 genes for qPCR validation. The expression patterns of UBC, PLA2R1, SMC5, PTPRO, PFN2, TIMM17B, NT5E and PCSK5 were consistent with bioinformatic predictions, supporting their potential roles in NPC recurrence. It is worth noting, however, that not all selected genes exhibited consistent expression across validation experiments. Discrepancies were observed in several genes, potentially attributable to technical variations inherent to qPCR, biological heterogeneity within clinical samples, or differences between cell line models and primary tissues. A notable example is UBC, which was significantly upregulated in cell lines but did not show a significant difference in clinical tissues. This specific discrepancy underscores the broader challenges in validation, which can be attributable to the biological heterogeneity within clinical samples, comprising a mixture of tumour, stromal, and immune cells, unlike homogeneous cell line models. Furthermore, for a multifunctional protein like ubiquitin C (UBC), whose activity is heavily regulated at the post‐translational level, mRNA abundance may not reliably reflect its functional state in the complex tumour microenvironment. These observations highlight the importance of interpreting validation results with caution and underscore the necessity of multi‐level experimental confirmation in subsequent studies.

Among the hub genes, UBC (Ubiquitin C) warrants special attention due to its central position in the PPI network. As a precursor of ubiquitin, its elevated expression in recurrent NPC and cell lines suggests a potential role in regulating protein stability and degradation via the ubiquitin‐proteasome system. We hypothesise that UBC overexpression may function as a driving oncogenic event in recurrence by promoting the degradation of key tumour suppressor proteins (e.g., components of the p53 signalling pathway, which is significantly enriched in our data), thereby enhancing tumour cell survival and treatment resistance. Alternatively, elevated UBC could be a consequence of cellular stress in aggressively relapsing tumours. Distinguishing between these possibilities and identifying its key substrate proteins in NPC represents exciting avenues for future research.

M‐type phospholipase A2 receptor 1 (PLA2R1) is a transmembrane glycoprotein which consists of a cysteine‐rich domain, fibronectin type II domain and eight carbohydrate recognition domains [54, 55, 56]. A limited number of studies supported that PLA2R1 was a tumour suppressor, and its expression was reduced in many cancers such as blast cancer, and leukaemia [57, 58]. Mechanistically, the classical tumour‐suppressive functional way of PLA2R1 was that the hypermethylation induced the activation of the kinase Janus‐kinase 2 (JAK2) and oestrogen‐related receptor a (ERRa)‐controlled mitochondrial proteins, and increased the accumulation of reactive oxygen species which led to apoptosis and senescence [59, 60]. However, the expression and functions of PLA2R1 mediated in NPC remain to be elucidated. Here, our results demonstrate that PLA2R1 is downregulated in recurrent NPC. Bioinformatic evidence suggests that this downregulation may be mediated by the miRNA hsa‐miR‐149‐5p, indicating a potential epigenetic mechanism for PLA2R1 suppression in NPC recurrence and metastasis. Thus, our data for the first time provide evidence for an important role of PLA2R1 in controlling the recurrence and metastasis of NPC.

Profilin 2 is an actin binding protein which could form an ATP‐actin‐PFN complex. PFN2 has been reported to be highly expressed in HNSC tissues and cell lines, where it promotes cell proliferation and metastasis via the activation of the PI3K/Akt/β‐catenin signalling pathway [61]. Meanwhile, four miRNAs (miR‐1‐3p, miR‐206, miR‐133a‐3p and miR‐133b) were commonly downregulated in HNSC, and confirmed to directly bind to regulate PFN2 [62]. In our study, we used the miRNA array datasets to construct the lncRNA‐miRNA‐mRNA regulated networks, then found that miR‐1‐3p and miR‐206 were identified in the relapse. Furthermore, results of the previous studies highlighted that the expression of PFN2 was significantly high, and it was co‐expressed with lncRNAs in various cancers. In SCLC, lncRNA LYPLAL1‐DT acts as an oncogenic lncRNA, which negatively regulates miR‐204‐5p leading to the upregulation of PFN2, promoting cell proliferation, migration and invasion [63]. In ESCC, the overexpression of lnc SBF2‐AS1 promoted the progression by repressing miR‐494 to upregulate PFN2 expression [64]. Therefore, we hypothesised that the overexpression of PFN2 may play an important regulatory role in the progression of relapse in posttreatment NPC.

Beyond their individual roles, our ceRNA network analysis places PLA2R1 and PFN2 within a broader regulatory context that may elucidate their contribution to NPC recurrence. While our network analysis did not identify specific lncRNAs sponging hsa‐miR‐149‐5p to regulate PLA2R1, existing literature documents that lncRNAs including LINC00467 and HOTAIR can function as molecular sponges for this miRNA [65, 66, 67]. This supports a plausible model wherein PLA2R1 downregulation in recurrent NPC may occur through similar ceRNA mechanisms. Concurrently, pathway analyses revealed significant alterations in p53 signalling and oxidative phosphorylation, suggesting that PLA2R1 suppression might facilitate tumour cell survival through diminished ROS‐induced apoptosis and senescence that mechanisms have been previously established in other cancers [57, 58, 60, 68], Similarly, our data provide concrete evidence for PFN2 regulation through identified ceRNA axes, including hsa‐miR‐4732‐5p sponged by AC016705 and STEAP3‐AS1, and hsa‐miR‐372‐3p sponged by STEAP3‐AS1. These interactions potentially drive PFN2 overexpression, which may promote proliferation and metastasis via PI3K/Akt/β‐catenin signalling which is consistent with GSVA results showing activated proliferation pathways [30, 31, 36, 37, 38, 40, 41, 59]. Notably, the concurrent enrichment of immune‐related pathways, including “B cell receptor signalling” and “cytokine‐cytokine receptor interaction,” suggests that PLA2R1 and PFN2 dysregulation may extend beyond cell‐autonomous effects to modulate immune microenvironment composition and function. This integrated perspective proposes a multi‐axial mechanism wherein ceRNA‐mediated regulation of PLA2R1 and PFN2 converges with metabolic reprogramming and immune modulation to drive NPC relapse, providing a compelling framework for future mechanistic investigation.

## 5 Limitations of the Study

Although this study identified potential ceRNA networks associated with nasopharyngeal carcinoma (NPC) recurrence, several limitations should be noted. First, the RNA‐seq analysis was based on a relatively small sample size (*n* = 3 per group), and the samples were collected from different hospitals and time periods, which may introduce bias and limit the generalisability of the findings. Although we employed stringent statistical thresholds and validated key results in independent models, the proposed ceRNA network should be viewed as a preliminary yet valuable framework. Future multi‐center studies with larger sample sizes are essential to confirm its robustness and generalisability. Second, the construction of the ceRNA network relied on computational predictions and expression correlations, albeit supplemented by experimentally validated miRNA‐target interactions from databases. Incorporation of additional evidence, such as CLIP‐seq data, could further strengthen the reliability of the predicted interactions in future studies. Finally, the lack of in vivo and in vitro functional experiments to mechanistically validate the proposed lncRNA–miRNA–mRNA regulatory networks represents another important limitation. Nonetheless, the findings of this study provide valuable resources and insights for the development of therapeutic strategies for recurrent NPC. Future multi‐center studies with larger sample sizes, together with experimental validation, are warranted to confirm and extend these results.

## 6 Conclusions

In this study, we systematically analysed the lncRNA‐mediated ceRNA network in posttreatment recurrent nasopharyngeal carcinoma (NPC) through whole‐transcriptome sequencing and bioinformatics approaches. We identified 813 mRNAs, 143 lncRNAs, and 24 miRNAs that constitute a relapse‐specific ceRNA network, with 23 hub mRNAs significantly associated with patient survival. Functional enrichment analyses revealed that these dysregulated RNAs are primarily involved in critical pathways such as the p53 signalling pathway, cell cycle regulation, oxidative phosphorylation, and immune‐related processes. Key genes, including UBC, PLA2R1, PTPRO, SMC5, PFN2, TIMM17B, NT5E and PCSK5, were experimentally validated in NPC cell lines and tissues, confirming their potential roles in recurrence. Our findings highlight the importance of the ceRNA regulatory network in NPC relapse, providing novel insights into the molecular mechanisms driving disease progression. These results suggest that targeting specific lncRNA‐miRNA‐mRNA interactions could offer new therapeutic strategies for managing recurrent NPC. Further functional studies are warranted to elucidate the precise mechanisms of these regulatory molecules and their clinical applications.

## Author Contributions

**Xiaoqin Fan and Guohui Nie:** conceptualisation, methodology, resources, writing – original draft, writing – review and editing, supervision, project administration, and funding acquisition. **Qihong Liu and Jingxiao Lu:** writing – original draft, investigation, formal analysis, data curation, validation, visualisation, writing – review and editing, and funding acquisition. **Zhiqiang Wang and Qingming Li:** investigation, formal analysis, data curation, validation, and visualisation. **Jianhui Wu and Kexing Lyu:** data curation, validation, and visualisation. **Beiping Miao and Wenbin Lei:** data curation, validation, and visualisation. All authors have read and agreed to the published version of the manuscript.

## Funding

The study was supported by the Shenzhen Science and Technology Innovation Committee (Project no. JCYJ20210324103005014, JCYJ20240813140521028, JCYJ20210324103007019); The Medical‐Engineering Interdisciplinary Research Foundation of ShenZhen University (Project no. 2023YG003); Futian Healthcare Research Project of ShenZhen (Project no. FTWS2021088), Shenzhen Otorhinolaryngology Clinical Research Center (Project no. 20220819120540004‐010). The funders had no role in study design, data collection and analysis, decision to publish, or preparation of the manuscript.

## Ethics Statement

This project was approved by the Ethics Committee of Eighth Affiliated Hospital of Sun Yat‐sen University (No. 2022‐029‐01). Written informed consent was obtained from all subjects.

## Consent

All patients provided written informed consent.

## Conflicts of Interest

The authors declare no conflicts of interest.

## Supporting information


**Appendix S1:** jcmm70973‐sup‐0001‐AppendixS1.xlsx.

**Figure S1:** The relapse specific differentially expressed mRNAs analysis. (A) Volcano map of differentially expressed mRNAs between the RelapseT and primaryT groups. (B) Heatmap of top 15 up/down‐regulated differentially expressed mRNAs between the RelapseT and primaryT groups; (C) Bubble graph of differentially expressed mRNAs enriched by GO analysis between the RelapseT and primaryT groups; (D) Bubble graph of differentially expressed mRNAs enriched by KEGG function analysis between the RelapseT and primaryT groups; (E) Venn diagram of the relapse specific significant up‐regulated mRNAs in the NPC; (F) Bubble graph of the 578 significant up‐regulated mRNAs enriched by KEGG analysis; (G) Venn diagram of the relapse specific significant down‐regulated mRNAs in the NPC; (H) Bubble graph of the 235 significant down‐regulated mRNAs enriched by KEGG analysis; (I) Survival plot of the 23 genes; (J) Survival plot of the UBC gene.

**Figure S2:** The relapse specific differentially expressed lncRNAs analysis. (A) Volcano map of differentially expressed lncRNAs between the RelapseT and primaryT groups. (B) Heatmap of top 15 up/down‐regulated differentially expressed lncRNAs between the RelapseT and primaryT groups; (C) Bubble graph of differentially expressed lncRNAs enriched by GO analysis between the RelapseT and primaryT groups; (D) Bubble graph of differentially expressed lncRNAs enriched by KEGG function analysis between the RelapseT and primaryT groups; (E) Venn diagram of the relapse specific significant up‐regulated lncRNAs in the NPC; (F) Venn diagram of the relapse specific significant down‐regulated lncRNAs in the NPC.

## Data Availability

The raw sequence data reported in this paper have been deposited in the Genome Sequence Archive in the National Genomics Data Center that is publicly accessible at https://ngdc.cncb.ac.cn/gsa‐human (HRA009977). Publicly available datasets (GSE102349; GSE118720; GSE32960; and GSE36682) were analysed in this study. All the datasets were obtained from the GEO (http://www.ncbi.nlm.nih.gov/geo) database.
