# Supplementary figures and images for: Integrated Analysis of the LncRNA‐Mediated ceRNA Network Associated With Prognosis in Posttreatment Recurrent Nasopharyngeal Carcinoma
Source: J Cell Mol Med. 2025 Dec 7;29(23):e70973. doi: 10.1111/jcmm.70973 (PMC12682473; doi:10.1111/jcmm.70973)

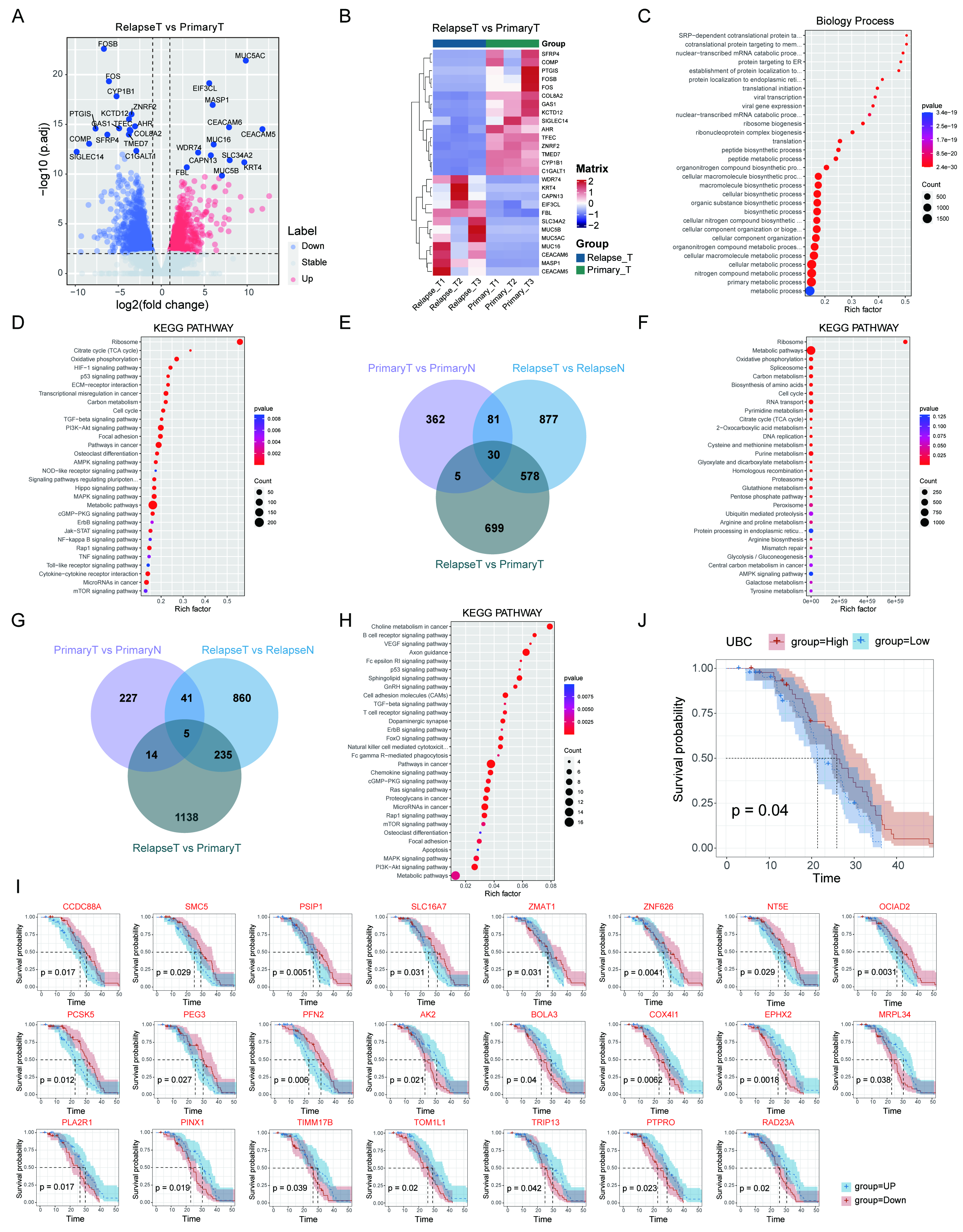

Supplement: Supplementary file 2 — Figure S1: The relapse specific differentially expressed mRNAs analysis. (A) Volcano map of differentially expressed mRNAs between the RelapseT and primaryT groups. (B) Heatmap of top 15 up/down‐regulated differentially expressed mRNAs between the RelapseT and primaryT groups; (C) Bubble graph of differentially expressed mRNAs enriched by GO analysis between the RelapseT and primaryT groups; (D) Bubble graph of differentially expressed mRNAs enriched by KEGG function analysis between the RelapseT and primaryT groups; (E) Venn diagram of the relapse specific significant up‐regulated mRNAs in the NPC; (F) Bubble graph of the 578 significant up‐regulated mRNAs enriched by KEGG analysis; (G) Venn diagram of the relapse specific significant down‐regulated mRNAs in the NPC; (H) Bubble graph of the 235 significant down‐regulated mRNAs enriched by KEGG analysis; (I) Survival plot of the 23 genes; (J) Survival plot of the UBC gene. [file JCMM-29-e70973-s003.tif]

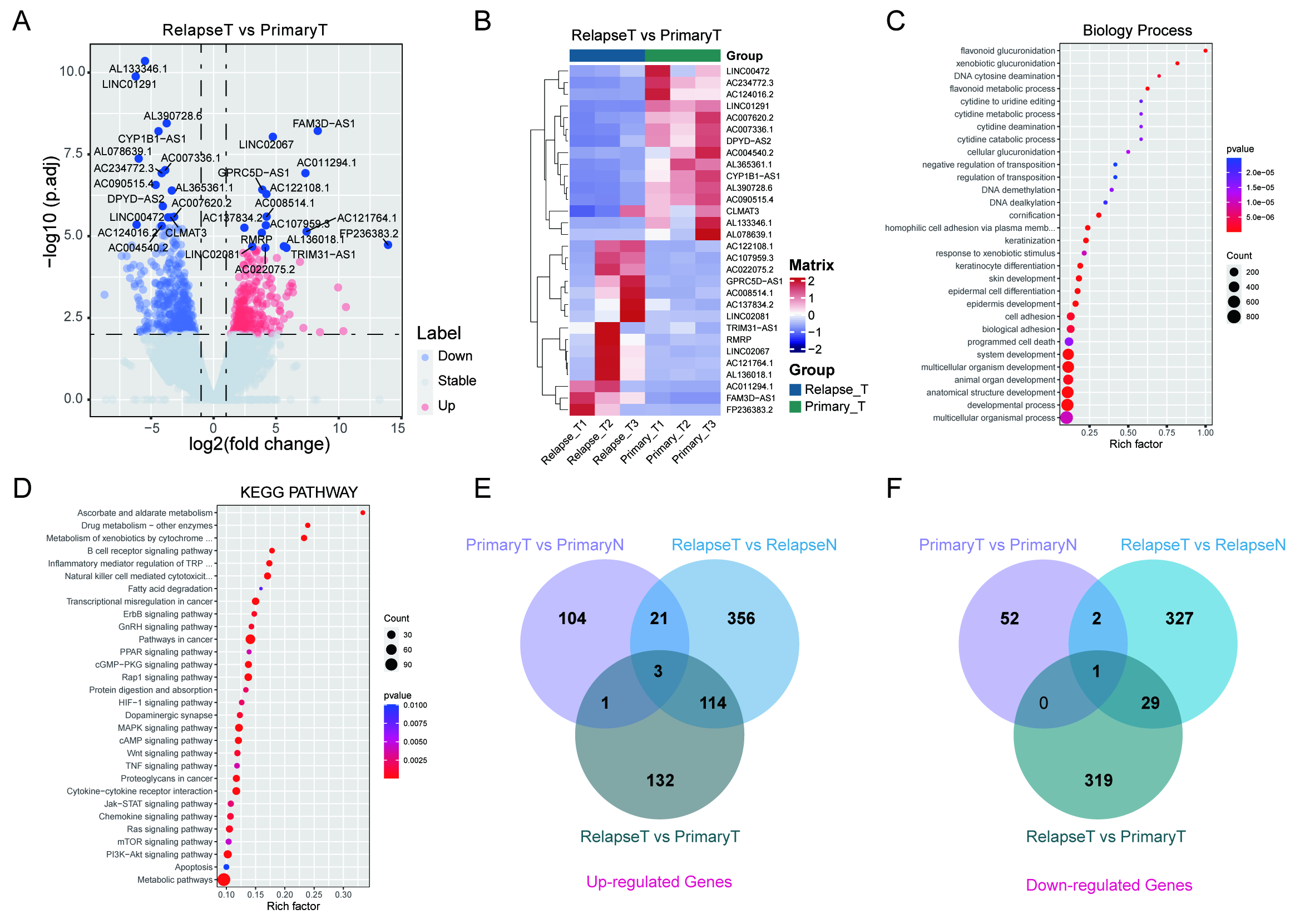

Supplement: Supplementary file 3 — Figure S2: The relapse specific differentially expressed lncRNAs analysis. (A) Volcano map of differentially expressed lncRNAs between the RelapseT and primaryT groups. (B) Heatmap of top 15 up/down‐regulated differentially expressed lncRNAs between the RelapseT and primaryT groups; (C) Bubble graph of differentially expressed lncRNAs enriched by GO analysis between the RelapseT and primaryT groups; (D) Bubble graph of differentially expressed lncRNAs enriched by KEGG function analysis between the RelapseT and primaryT groups; (E) Venn diagram of the relapse specific significant up‐regulated lncRNAs in the NPC; (F) Venn diagram of the relapse specific significant down‐regulated lncRNAs in the NPC. [file JCMM-29-e70973-s001.tif]
